# Genome-Wide Prediction of Transcription Start Sites in Conifers

**DOI:** 10.3390/ijms23031735

**Published:** 2022-02-03

**Authors:** Eugeniya I. Bondar, Maxim E. Troukhan, Konstantin V. Krutovsky, Tatiana V. Tatarinova

**Affiliations:** 1 Laboratory of Forest Genomics, Institute of Fundamental Biology and Biotechnology, Siberian Federal University, 660036 Krasnoyarsk, Russia; ebondar@sfu-kras.ru; 2 Laboratory of Genomic Research and Biotechnology, Federal Research Center “Krasnoyarsk Science Center” Siberian Branch, Russian Academy of Sciences, 660036 Krasnoyarsk, Russia; 3 Persephone Software LLC, Agoura Hills, CA 91301, USA; mtroukhan@persephonesoft.com; 4 Department of Forest Genetics and Forest Tree Breeding, Georg-August University of Göttingen, 37077 Göttingen, Germany; 5 Center for Integrated Breeding Research, Georg-August University of Göttingen, 37075 Göttingen, Germany; 6 Laboratory of Population Genetics, N. I. Vavilov Institute of General Genetics, Russian Academy of Sciences, 119333 Moscow, Russia; 7 Scientific and Methodological Center, G. F. Morozov Voronezh State University of Forestry and Technologies, 394087 Voronezh, Russia; 8 Department of Genomics and Bioinformatics, Institute of Fundamental Biology and Biotechnology, Siberian Federal University, 660074 Krasnoyarsk, Russia; 9 Department of Biology, University of La Verne, La Verne, CA 91750, USA; 10 Functional Genomics Group, N. I. Vavilov Institute of General Genetics, Russian Academy of Sciences, 119333 Moscow, Russia; 11 A. A. Kharkevich Institute for Information Transmission Problems, Russian Academy of Sciences, 127051 Moscow, Russia

**Keywords:** transcription start site, transcription factor binding site, TATA-box, conifer, gymnosperms, promoter prediction

## Abstract

The identification of promoters is an essential step in the genome annotation process, providing a framework for gene regulatory networks and their role in transcription regulation. Despite considerable advances in the high-throughput determination of transcription start sites (TSSs) and transcription factor binding sites (TFBSs), experimental methods are still time-consuming and expensive. Instead, several computational approaches have been developed to provide fast and reliable means for predicting the location of TSSs and regulatory motifs on a genome-wide scale. Numerous studies have been carried out on the regulatory elements of mammalian genomes, but plant promoters, especially in gymnosperms, have been left out of the limelight and, therefore, have been poorly investigated. The aim of this study was to enhance and expand the existing genome annotations using computational approaches for genome-wide prediction of TSSs in the four conifer species: loblolly pine, white spruce, Norway spruce, and Siberian larch. Our pipeline will be useful for TSS predictions in other genomes, especially for draft assemblies, where reliable TSS predictions are not usually available. We also explored some of the features of the nucleotide composition of the predicted promoters and compared the GC properties of conifer genes with model monocot and dicot plants. Here, we demonstrate that even incomplete genome assemblies and partial annotations can be a reliable starting point for TSS annotation. The results of the TSS prediction in four conifer species have been deposited in the Persephone genome browser, which allows smooth visualization and is optimized for large data sets. This work provides the initial basis for future experimental validation and the study of the regulatory regions to understand gene regulation in gymnosperms.

## 1. Introduction

Transcription is a mechanism of information transmission encoded in protein-coding genes conducted by RNA Polymerase II, resulting in the production of messenger RNAs (mRNAs). This process is subject to complex regulation via binding of transcription factors (TFs) to appropriate genomic sites consisting of regulatory nucleotide motifs typically located within the 1000 bp region upstream of the transcription start sites (TSSs). This region is called the promoter. The TSS position corresponds to the first nucleotide transcribed by RNA Pol II. Eukaryotic genes can have multiple alternative TSSs [1,2].

The core promoter is a stretch of DNA up to 250 bp long located immediately upstream of the TSS and required for transcription initiation. There are two types of transcription initiation: narrow, generally associated with the regulation of tissue-specific and stress-response genes, and broad, typically occurring in housekeeping genes under a constitutive expression pattern [3]. The corresponding area is called a transcription start region (TSR) when the transcription initiation region is broad. The best-known regulatory motif in core promoter regions is the TATA-box, a recognition site for the TATA-binding protein (TBP). This motif has a highly conserved consensus sequence TATA(A/T)A(A/T) found in 5–60% of all RNA Pol II promoters [1,4,5,6,7,8]. Another common motif is Inr (initiator) with the consensus sequence YYA+1NT/AYY, which occurs at the start of transcription. Inr is more widespread than any other sequence motif [3] and is commonly found in housekeeping genes, whose transcription is initiated not with a single start but with positional clusters of TSSs referred to as TSRs [9]. In contrast, TATA-containing promoters are narrower and associated with tissue- or context-specific gene expression [10]. Other common core promoter elements are the TFIIB recognition element (BREu, consensus G/CG/CG/ACGCC, and BREd, consensus G/ATT/AT/GT/GT/GT/G [11,12]), the downstream promoter element (DPE, consensus RGWYV [13,14]), and the downstream core element (DCE, consensus CTTC, CTGT, AGC [15]).

The identification of promoters is a crucial step in genome annotation, providing a framework for understanding gene regulatory networks and their role in transcription regulation [8]. In recent years, high-throughput methods for identifying TSS and TFBS have advanced considerably. Such techniques as chromatin immunoprecipitation combined with microarray or sequencing analysis (ChIP-chip and ChIP-seq, respectively), identification of DNase I-hypersensitive sites (DHS), cap analysis of gene expression (CAGE), and paired-end analysis of TSS (PEAT) have allowed the accumulation of a substantial amount of data on plant regulatory regions [16,17]. TF studies in agriculturally important species, such as the *Prunus* genus, have utilized numerous resources and techniques, including gene expression analysis of different agronomic traits, quantitative RT-PCR, cDNA-AFLP, LC-ESI-MS, RNA, and DNA blotting, to build a database of genus-specific TFs and to provide a comprehensive source for further functional studies and breeding programs [18]. However, those experimental methods of identifying the TSS and promoter regions are time-consuming, labor-intensive, and expensive. Several computational approaches have been developed to provide fast and accurate ways to predict the location of TSSs and regulatory motifs on the whole-genome scale. These include Bayesian classification based on positional densities of oligonucleotides for detecting TSS in human genomic sequences [19], neural networks for predicting TSS in plant promoters [20,21], and conditional random fields for identifying TSS in eukaryotic promoters [22]. Strategies for genome-wide discovery of novel cis-regulatory motifs using position weight matrices (PWMs) and expression data were successfully implemented for rice and *Arabidopsis* [6,23], hop [24], and grapevine [25]. Genome-wide analysis of core promoter elements using PWMs and orthologous-based prediction were performed for several monocot and dicot species by Kumari and Ware [26].

It has been shown that promoters differ from the rest of the genome in several measurable properties: low DNA stability, high bendability, curvature, etc. [7,27,28,29]. DNA stability is associated with the melting of the double-stranded molecule before transcription initiation and is commonly calculated as the standard free energy of a DNA duplex. It has been implemented successfully for promoter identification in various eukaryote species [30]. Numerous studies have reported a curved DNA region upstream of TSSs and higher bendability of the area that interacts with DNA binding proteins [28,31,32,33]. Other features of promoter regions include CpG islands, GC-skew, and decreased genetic variability [7,29,34]. The excess of Cs over Gs (GC skew) in the sense strand around TSSs was reported for several plant species [35,36] and metazoans [37]. The peak of the GC skew around TSSs can be explained by cytosine deamination during the transcription due to RNA polymerase preferential protection of nucleotides on the non-transcribed strand [35].

It may seem surprising, but the properties of coding and promoter regions are correlated. The frequency of guanine and cytosine nucleotides at the third position GC_3_ is one of the critical properties of coding regions. Nucleotides at the third position are less subjected to selection than at the first two due to the degeneracy of a genetic code. It was observed that based on GC_3_, the genomes could be classified into those having unimodal and bimodal GC_3_ distributions. For instance, all currently sequenced grass genomes have a bimodal distribution of GC_3_, while the CDS of dicot plants shows a unimodal distribution of GC_3_ [38,39]. It was previously thought that bimodal GC_3_ is a specific feature of grass genomes. Later, it was demonstrated that GC content in other monocot species, such as *Curcuma longa, Zingiber officinale, Elaeis guineensis*, and *Zantedeschia aethiopica*, also exhibits a bimodal GC_3_ distribution [40,41,42]. It has been shown that genes with a higher GC_3_ content also have a higher frequency of TATA-boxes and are more likely to be stress-related [39].

Conifers are an ancient group of dicot plants represented by more than 600 species that play a significant role in boreal forest ecosystems. Due to their enormous size and highly repetitive nature, deciphering conifer genomes takes more time and effort than many other plant species. Several mega-genomes of conifer species have been sequenced and assembled to the draft state recently, resulting in multiple contigs and gaps in chromosome coverage. Although such annotations are preliminary, they provide an opportunity for structural and functional analysis. Even an incomplete genome contains keys to understanding regulatory relationships between genome elements, and this analysis requires knowledge of the precise locations of promoter sequences.

The aim of this study was to enhance and expand the existing genome annotations by predicting TSSs for the four recently published conifer species: loblolly pine (*Pinus taeda* L.), white spruce (*Picea glauca* (Moench) Voss), Norway spruce (*Picea abies* (L.) H. Karst.), and Siberian larch (*Larix sibirica* Ledeb.). Siberian larch is a cold-resistant fast-growing tree known for its rot-resistant timber, making it especially valuable in construction. Norway spruce had the first genome sequenced among gymnosperms. It is widely cultivated as an ornamental tree worldwide and is a source of timber for paper and construction lumber production. White spruce is another cold-resistant tree native to northern parts of North America with tremendous economic value in Canada. Loblolly pine has one of the largest genome sizes (22 Gbp) among sequenced plant species and is considered one of the most significant sources of timber in the U.S.

## 2. Results

### 2.1. Prediction of TSS in Four Conifer Species

Alignment of RNA-seq, ESTs, and RefSeq protein to the four genomes data allowed the identification of 9260 evidence-supported gene models for *Pinus taeda*, 16,853 for *Picea glauca*, 7587 for *Picea abies*, and 23,077 for *Larix sibirica* (Table 1, Supplementary Figure S1). For promoter prediction, we used TSSPlant [20], which utilizes neural networks to estimate up to 17 features, such as the presence of classic motifs, nucleotide composition variation, and others (more details in Methods or [20]). The use of TSSPlant generated predictions of 22,633 TSS positions in *P. taeda*, 25,889 in *P. abies*, 44,651 in *P. glauca*, and 62,420 in *L. sibirica*. From 13.3% to 14.3% of identified TSS positions occurred within the coding parts of their respective gene models (Figure S2) and were excluded.

To select the most likely TSS among the multiple predictions for a given gene, we compared the length of each 5′ UTR to the distribution of 5′ UTR lengths in four plant species, two dicots, *A. thaliana* and *P. trichocarpa*, and two monocots, *O. sativa* and *S. bicolor* (Figure S3A,B). Two parameters, *k* and *theta*, which determine the shape and scale of the gamma distribution, were computed as follows: *theta = v/m, k = m/theta*. Using *k* = 0.62 and *theta* = 238.99, we selected predictions that better fit the theoretical 5′ UTR length distribution (Figure S3C). After filtering out predictions within the respective coding regions and selecting the highest-scoring positions, 10,367 *P. abies*, 16,629 for *P. glauca*, 9149 for *P. taeda*, and 23,016 for *L. sibirica* were identified as putative TSSs (Table 1). All gene models with corresponding predicted TSSs were deposited in the Persephone genome browser and are available at https://web.persephonesoft.com (accessed on 31 January 2022).

Genome annotations of the Siberian larch and white spruce were performed with the MAKER [47] pipeline using transcriptome data and available ESTs and RNA-seq data for these and other closely related species. Therefore, it became possible to conduct an automated prediction of 13,228 UTRs for *L. sibirica* and 14,056 UTRs for *P. glauca* based on the available ESTs within the annotation pipeline. We compared the TSSs predicted by the maker pipeline and TSSs predicted by the TSSPlant algorithm with filtering based on the 5′ UTR length distribution of model plant species. We showed that the positional distribution of TSSs predicted by the de novo method was similar to that predicted using RNA support (Figure S4).

**Figure 1:**
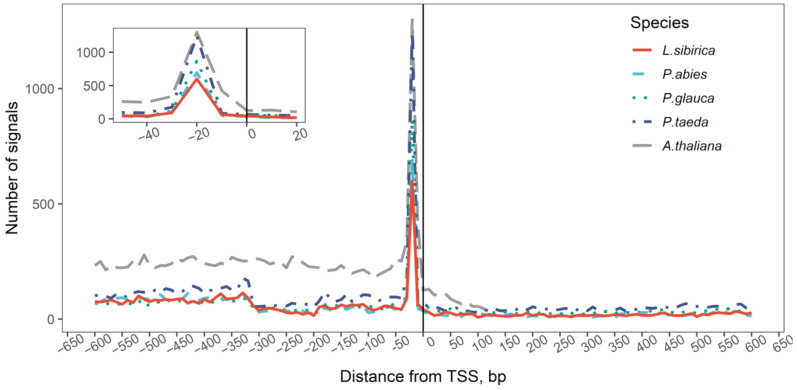
Frequency of the TATA(A/T)A(A/T) motif in the TSS-centered promoter region.

In the predicted promoters, the occurrence of the TATA(A/T)A(A/T) motif shows a pronounced peak approximately 20 bp upstream of the predicted TSS position for all four species (Figure 1), which corresponds well to the canonical location of the TATA-box, since 30 to 50% of eukaryotic promoters contain a TATA-box 40 to 15 bp upstream of the TSS.

When comparing the number of promoters containing TATA-box or CA initiator motif, approximately half of the analyzed sequences (46–53%) had CA motif within the area [−2; +2] around the TSS, while TATA-box was found in 5–8% of promoters in the [–40; –20] region relative to the TSS. Among TATA-containing promoters, approximately half of them (1.6–2 ratio of TATA-containing to TATA-and-CA-containing) contained both TATA and CA motifs (see more in Table S3 and Figure S5).

**Figure 2:**
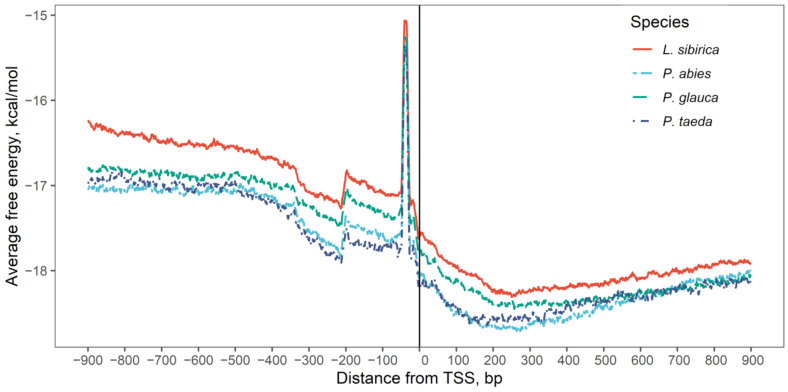
Distribution of DNA free energy around TSS position predicted by TSSPlant.

Change in the standard free energy of a DNA duplex across the genome sequence is a strong indicator of a promoter region and has been implemented successfully for promoter prediction. We used this as supporting evidence for promoters predicted by TSSPlant. The free energy profile shows a peak around –40 bp and a sharp decline around putative TSS (Figure 2).

**Figure 3:**
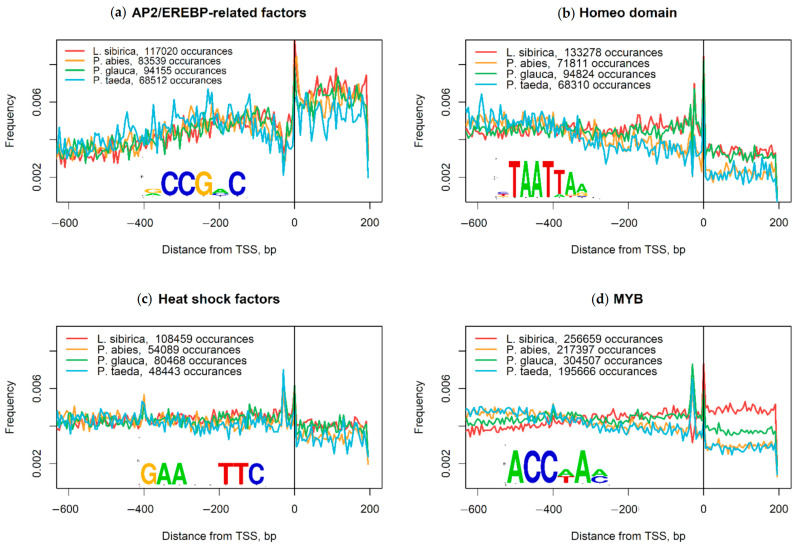
Positional distribution of transcription factor binding sites (TFBS) in *Larix sibirica*, *Picea abies*, *Picea glauca*, and *Pinus taeda* based on PWM scanning using TRANSFAC. (**a**) AP2/EREBP-related factors; (**b**) Homeodomain; (**c**) Heat shock transcription factors; (**d**) Myb transcription factors.

To determine the positional distribution of TFBS, we scanned the identified promoter regions for the presence of several developmental and stress-related TFBS using TRANSF AC and MATCH. PWMs that belong to the Homeodomain, Heat shock, and Myb TFs show two peaks in their positional distribution (Figure 3b–d), while AP2/EREBP TFs have an apparent decrease near the TATA-box region (Figure 3a).

**Figure 4:**
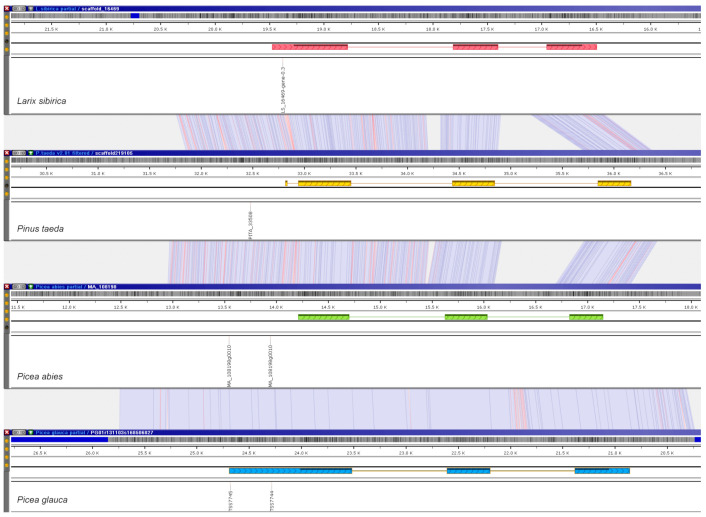
Orthologous genes of FLORICAULA/LEAFY-like proteins in *L. sibirica*, *P. taeda*, *P. abies*, and *P. glauca* with corresponding predicted TSS positions (depicted by the vertically-oriented labels) in their upstream regions are aligned using the genome browser Persephone. Red, yellow, green, and blue boxes represent exons. Light blue ribbon-like connectors indicate identical areas, blue lines mark nucleotide substitutions, and red lines indicate indels. The visualization is available at https://web.persephonesoft.com/?bookmark=43C6DEFD15C23F5F40A8AFF25F844042 (accessed on 31 January 2022).

Additionally, the promoters of two developmental genes, FLORICAULA/LEAFY and WLIM2a, were scanned for the presence of conserved sequence motifs. For orthologs of LEAFY, a helix-turn-helix transcription factor regulating inflorescence development in many flowering plant species [48], there are two predicted TSS positions (Figure 4) in the upstream regions of *P. abies* and *P. glauca*, and one prediction for *P. taeda* and *L. sibirica*.

**Figure 5:**
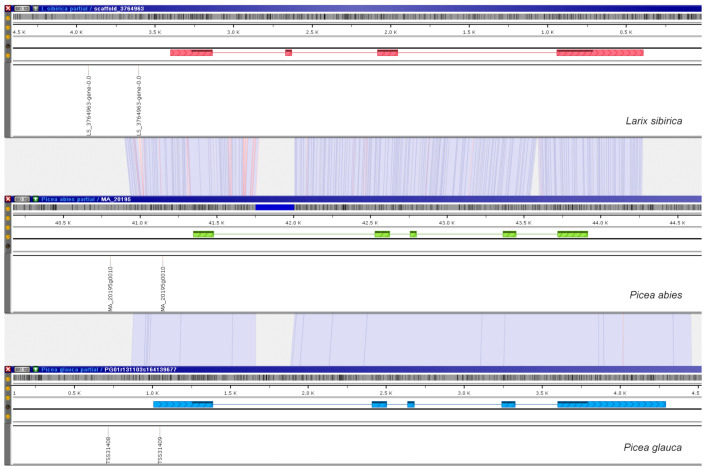
Orthologous genes of WLIM2a in *L. sibirica*, *P. abies*, and *P. glauca* with corresponding predicted TSS positions (depicted by the vertically-oriented labels) in their upstream regions. Red, green, and blue boxes represent exons. Light blue ribbon-like connectors indicate identical areas, blue lines mark nucleotide substitutions, and red lines indicate indels. The visualization is available at https://web.persephonesoft.com/?bookmark=4239E3155493E8E21C61A9932BD502EE (accessed on 31 January 2022).

For LIM domain-containing WLIM2a, a regulatory protein that triggers the formation of actin bundles playing an essential role in actin cytoskeleton organization [49], three orthologs were found in *L. sibirica*, *P. abies,* and *P. glauca*, each of which had two predicted TSS sites (Figure 5).

### 2.2. Nucleotide Composition Analysis of Promoter and Coding Sequences

**Figure 6:**
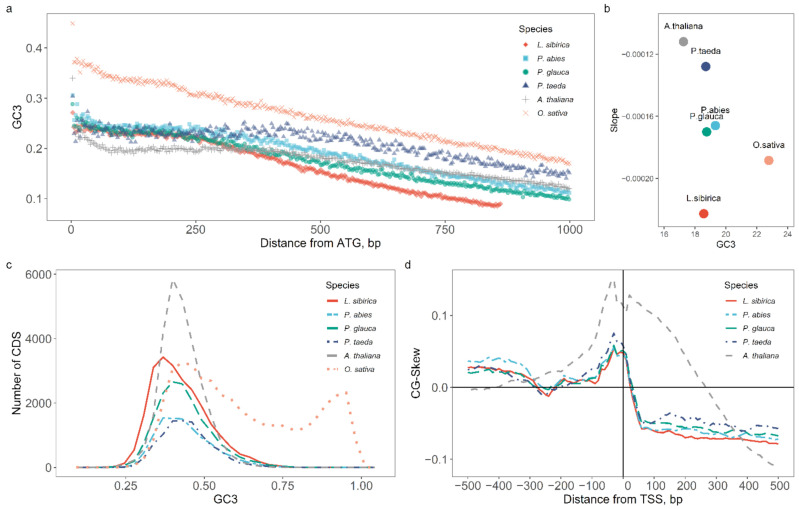
Some GC statistics for four conifer species, *Larix sibirica, Picea abies, Picea glauca, Pinus taeda*, and two model plant species, *Arabidopsis thaliana* and *Oryza sativa*: (**a**) GC_3_ gradient of coding sequences, (**b**) GC_3_ gradient slope, (**c**) GC_3_ distribution across all CDSs, (**d**) CG-skew around TSSs.

We computed GC3 for all coding regions retrieved from current annotations and had RNA-SEQ support. Similar to other dicot plants, conifers possess a unimodal GC_3_ distribution, with a mean of 0.43 (sd = 0.087, Figure 6c). Analyzing coding sequences in several plant species has indicated a GC_3_ gradient from the 5′ to 3′ end of a gene [39,50]. All four analyzed species had a similar GC_3_ gradient that gradually decreased 250 bp after the start of the coding sequence (Figure 6a). We divided the genes into GC_3_-poor and GC_3_-rich categories using 10% and 90% quantiles of GC_3_ to define the GC_3_-rich and GC_3_-poor gene sets. We determined the relationship between the position of the codon in the coding sequence and the GC_3_ content for both GC_3_ categories, applying linear regression to the first 1000 nucleotides of the coding sequence (Figure 6b). In Siberian larch and rice, the slope of the regression line is more prominent than in loblolly pine and thale cress. These results agree with a previous report on GC distribution patterns in gymnosperms [41].

Similar to *A. thaliana* and *O. sativa* [35,36], the CG-skew in the four examined conifer species exhibited a distinct peak around the TSS (Figure 6d). The height of the peak in the four conifer species is lower than that in the thale cress. It can be due to biological differences or the lower quality of genome assembly.

**Figure 7:**
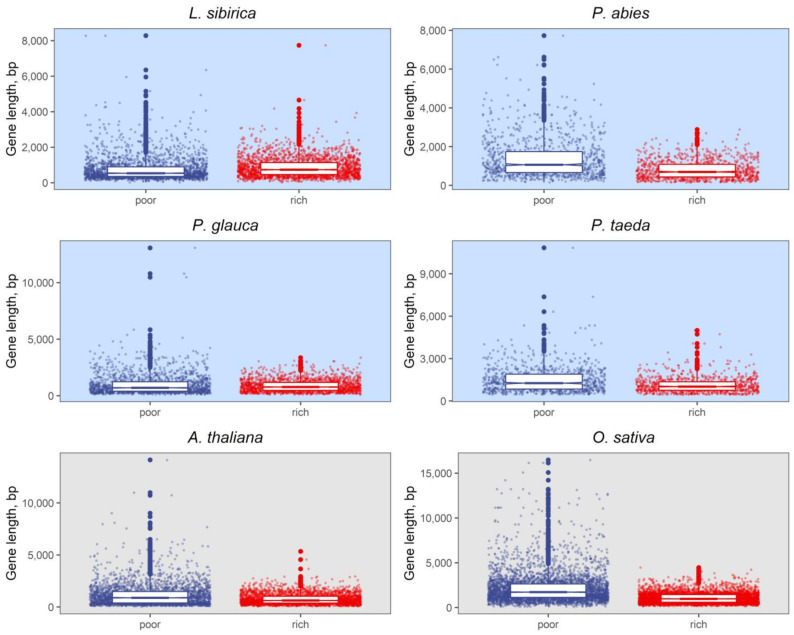
The difference in coding sequence length between GC_3_-poor and GC_3_-rich genes; 10% and 90% quantiles were used to divide genes into GC_3_-poor and GC_3_-rich classes (blue and red, respectively).

To test whether the difference in gene length between GC_3_-poor and GC_3_-rich genes can be observed in gymnosperm genomes, we compared these two classes of genes (Figure 7 and Figure S6). A Mann–Whitney U test indicated that the CDS length in GC_3_-poor genes was significantly longer than that in GC_3_-rich genes (2.20 × 10^−16^ < *p* < 6.09 × 10^−12^, see Table S2).

**Figure 8:**
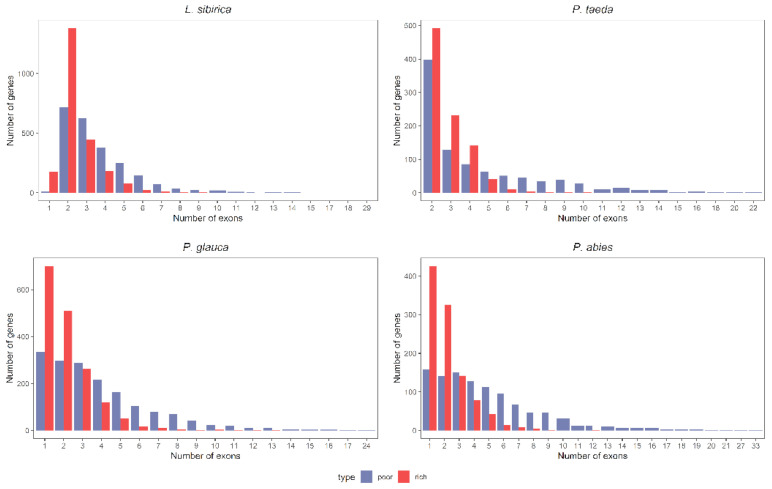
Distribution of the exon number per gene in GC_3_-poor and GC_3_-rich genes in *L. sibirica*, *P. abies, P. glauca*, and *P. taeda*. The number of genes in the GC3-poor and GC3-rich categories was the same within each organism.

There was a significant difference in the number of exons between the two classes of genes; this trend holds for all studied gymnosperms and angiosperms. According to the current genome annotations, GC-rich genes tend to have between two and four exons in Siberian larch and loblolly pine and two or fewer exons in Norway and white spruces (Figure 8). Genes with more than five exons are generally GC-poor in all species.

## 3. Discussion

While extensive studies on mammalian regulatory regions have been conducted, plant promoters were left out of the limelight and have remained poorly investigated. Promoter prediction programs have been mainly developed for and trained on a limited range of model organisms such as humans, *Drosophila*, thale cress, or rice. Nevertheless, the existing annotations for sequenced conifer genomes allow for computational prediction of biologically relevant elements, such as TSS and TFBS, and meaningful comparative analysis.

### 3.1. Prediction of TSS

Plants rely on TATA-box to initiate the transcription of most genes. The TATA-box is located approximately 40 bp upstream of the TSSs in the conifers. Although the most common location for the TATA-box is from 20 to 40 bp upstream of the TSSs, it has been previously reported that in some plants, such as *Vinis vinifera*, the TATA-box was observed within −70 bp relative to the TSSs [26]. The height of the peak of TATA frequency (Figure 1) directly measures the accuracy of the TSS prediction.

In addition to the TATA-box, a standard plant core promoter model [51] includes the initiator motif at the TSSs, AGGA-box (YA2-5KNGA2-4YY, ~80 bp upstream of the TSSs, [51,52]), and the downstream promoter element, DPE (RGWYVT, ~28–32 bp downstream from the TSSs, [51]). There are crucial differences in the promoter organization of plants and animals. Mammalian promoters commonly have a CAAT-box [53] instead of the plant AGGA. The TATA-box appears in less than 10% of mammalian promoters. The DPE motif typically occurs in TATA-less promoters; however, TATA and DPE may co-occur [51]. Animal promoters may also have BRE and motif ten (MTE) elements not found in plant promoters [53]. It has been reported that genes may have bidirectional promoters [54,55]; this feature has been thoroughly studied in mammals and less extensively in plants. Morton et al. [17] analyzed *A. thaliana* root samples to find precise TSS locations. They found that most promoters were TATA-less but contained a specific combination of transcription factor binding sites regulating gene expression. Yamamoto et al. [56] studied core promoters in *A. thaliana*, such as TATA and GA. They observed that the promoter architecture was related to the gene structure. The length of the 5′ UTR (a distance from the TSS to the start of translation) is also negatively correlated with the expression level of respective genes [39].

One of the standard computational strategies in predicting transcriptional targets in promoter regions is to search for a consensus model of a TF binding site (TFBS). Such consensus sequences are derived from experimentally identified binding sites and stored as a position weight matrix (PWM), which can be used to scan a sequence of interest. Several curated databases provide collections of PWMs, such as JASPAR [57], PlantRegMap [58], or TRANSFAC [59]. Despite the continuous improvement in PWM matching methods, they yield many false-positive predictions. Short length and high variability of the actual TF binding motifs cause the majority of matrix matches to miss functional binding sites (a so-called “futility theorem” [60]). However, PWM scanning can serve as a useful preliminary step in generating a list of candidate TFBSs that can be further filtered using other methods. Motif conservation across homologs, identification of overrepresented motifs in co-expressed genes, or clustering of multiple closely positioned motifs (cis-regulatory modules, CRMs) can be among these filters. The consistency in the location of specific motifs can also indicate an accurately predicted promoter.

The AP2/EREBP superfamily is one of the largest plant TFs, and it can be classified into three families: the AP2, RAV, and ERF (ethylene response factor) families. The most abundant ERF family is further divided into ERF and DREB subfamilies. ERF TFs can bind to a GCC-box element (AGCCGCC) and are involved in hormone signaling pathways and the regulation of pathogenesis-related genes. DREB TFs bind to the dehydration-responsive element (DRE) with the A/GCCGAC motif and regulate the expression of stress-responsive genes [61,62]. Homeodomain TFs are widely conserved proteins accounting for approximately 15–30% of all TFs in eukaryotes that drive the transcription of genes responsible for cell differentiation, morphogenesis, and stem cell pluripotency maintenance. They possess a DNA-binding domain containing a helix-turn-helix (HTH) structure that recognizes a short 5′-TAAT-3′ motif with very moderate specificity [63,64]. Cold, salinity, drought, and other protein-damaging stress factors induce activation and trimerization of HSF, allowing binding of each HSF monomer to a heat shock element (HSE). HSE is located at the TSS of HSP genes and includes at least two inverted repeats with a 5′-nGAAn-3′ (5′-nGAAnnTTCn-3′) consensus motif upstream of the TATA-box [65,66]. MYB-like proteins control plant metabolism, development, cell fate, and stress response. TFs containing the R2R3-type MYB domain, typical for plants, usually bind to an AC-enriched DNA motif (AC-elements), such as 5′-ACC(A/T)A(A/C)-3′ [67,68]. PWMs belonging to Homeodomain, Heat shock, and Myb TFs have two peaks (Figure 2b–d) that match AT-rich positions at 0 and –40 bp corresponding to TSS and TATA-box, respectively. Similarly, AP2/EREBP TFs with their GC-rich binding motifs had a pronounced drop near the TATA-box region (Figure 2a).

TSSPlant [20] combines the EM algorithm and neural networks to estimate a comprehensive set of features. The prediction accuracy is similar to another classic method that calculates the free energy of the free-energy change of the DNA duplex implemented in PromPredict [69]. To the best of our knowledge, there are practically no experimental studies verifying 5′-UTR on a genome-wide level or for a large subset of genes in conifers. However, experiments with individual genes have been carried out in *Picea glauca* [70,71,72]. A trans-activation assay with *Agrobacterium* transient transformation method was used to evaluate promoter–TF interactions in 12 genes involved in lignin biosynthesis enzymes, cell-wall synthesis and remodeling, and transcriptional regulation [70]. The promoter of cellulose synthase gene PgCesA3, containing MYB cis-regulatory elements [71], and cinnamyl alcohol dehydrogenase (CAD), containing cis-elements matching MYB, WRKY, and bHLH [72], have been confirmed to induce expression of GUS reporter gene in transgenic spruce in differentiating xylem and foliar guard cells, and in lignifying tissues, respectively. We suggest that on the datasets coming from non-model plant organisms, these two algorithms can be used together for higher accuracy of the TSS prediction. The results obtained in this study can be further validated in the future using chromatin immunoprecipitation analysis (ChIP-chip and ChIP-seq), identification of DNase I-hypersensitive sites, CAGE sequencing, or PEAT. In addition, the in silico predictions may be used as complementary support in cases of ambiguous signals that can be captured by ChIP-seq or CAGE-seq analysis.

### 3.2. Nucleotide Composition of Promoters and Coding Regions

Most angiosperm genomes have a distinct 5′ to 3′ decreasing gradient of the GC content of coding regions. This effect is manifested the most at the third codon position. There is a possible connection between recombination and the 5′–3′ GC gradient, as the recombination rate is higher around the TSSs, which creates a 5′–3′ recombination gradient [73,74]. It was proposed that a 5′–3′ GC gradient can indicate recombination initiation at TSSs [40].

Enrichment of DNA in guanine and cytosine nucleotides is associated with higher gene compactness and density and higher recombination rates than less GC-enriched regions [40]. It has been observed in multiple species that genes can be grouped into two classes based on the GC content in the third nucleotide position of the coding sequences [75,76,77]. As was reported by Serres-Giardi et al. [41] and Tatarinova et al. [39], in some plants, GC_3_-poor and GC_3_-rich genes differ significantly in length, with longer coding sequences tending to have a lower frequency of G+C nucleotides in the third position. It is also believed that the prevalence of GC nucleotides in shorter genes is the result of their length, as the GC content of a gene is an average of the existing GC gradient. The shorter GC-rich genes tend to be either mono-exonic or have fewer exons and introns in general, directly affecting the average GC content, as introns have a lower GC content than exons. According to Glémin et al. [40], the unimodal distribution of GC_3_ content indicates a smaller GC gradient within the genes and a lower recombination rate. It was also previously noticed that GC_3_-rich genes could show more variable expression, more frequently have TATA-dependent promoters, and are commonly involved in stress response pathways. An observed peak in the CG skew around the predicted TSSs (Figure 6d) had been previously linked to the transcriptional efficiency and methylation status of the GC_3_-rich genes [42].

The CG-skews in the four conifer genomes were lower than that reported for thale cress (*A. thaliana*), but to conclude whether it was due to the quality of conifer genome assembly or due to differences between gymnosperms and angiosperms (or monocots and dicots for that matter) many more genomes should be analyzed, which is beyond the scope of this study. However, it can be done using the tools presented here and will be an exciting avenue for prospective studies.

## 4. Materials and Methods

### 4.1. Genome Assemblies and Annotations

*Pinus taeda* genome assembly and annotation Pita v2_01 [45,78,79] were taken from https://treegenesdb.org/FTP/Genomes/Pita/v2.01 (accessed on 21 January 2020). Genome assembly PG29 v3.0 and the corresponding annotation for *Picea glauca* [44,80] were taken from ftp://plantgenie.org/Data/ConGenIE/Picea_glauca/PG29/v3.0/ (accessed on 16 January 2020); to our knowledge, assembly v4.0 has not yet been fully annotated. However, we also considered the manual annotation for assembly v4.0 added to annotation v3.0 *Picea abies* [43]; genome data Pabies_v1.0 were retrieved from ftp://plantgenie.org/Data/ConGenIE/Picea_abies/v1.0 (accessed on 16 January 2020). Corresponding annotations for the High Confidence set (predicted gene models with more than 70% homology with reference proteins) and the Medium Confidence set (predicted gene models with between 30% and 70% homology with reference proteins) as provided by the authors were grouped. For *Larix sibirica* [46], genome assembly under NCBI GenBank accession NWUY0000000000 (BioProject PRJNA393226) and unpublished draft annotation data were used. The detailed parameters of the genome assemblies used in the study are presented in Table 1.

### 4.2. Gene Filtering

To filter out possible pseudogenes and putative predicted coding sequences that do not represent functional genes, all gene models retrieved from genomic annotations were aligned against the database of RNA-seq data, including ESTs and TSAs (Table S1) of a related species using HISAT2 [81] (Table S4). To verify the selected gene models, we aligned the corresponding protein products to RefSeq plant protein sequences using BLASTp (Table S4).

### 4.3. Prediction of TSS

Prediction of putative TSSs was performed in the upstream sequences of selected genes, which were defined as regions of –1000 and +250 bp around the start codon, using the TSSPlant program [20]. TSSPlant utilizes the expectation-maximization (EM) algorithm and neural networks to estimate 17 and 15 features for predicting TATA and TATA-less promoters, respectively. The complex set of estimated parameters includes such features as the presence of classic plant promoter motifs (TATA, INR, DPE, YP), variation in nucleotide composition (CG- and AT-skews), oligomer scoring, and others. As the algorithm determines several possible start sites with the best scores, selecting the best prediction was utilized to leave one TSS per gene. Assuming that a gamma distribution can describe the length of the 5′-UTR, we compiled the pool of 5′-UTR lengths from the annotations of several model plants (*Arabidopsis thaliana*, *Oryza sativa*, *Sorghum bicolor*, and *Populus trichocarpa*). We computed k and theta parameters that determine the shape and scale of the distribution of the 5′-UTR lengths. The probability density function was used to select the most likely TSS positions using these parameters (Table S4).

### 4.4. Nucleotide Composition Analysis

Nucleotide frequency analysis of promoters was performed in TSS-centered sequences (–1000, +200 around TSS). CA and TATA motif frequencies were calculated with a sliding window (width = 20, increment step = 10) using the *stringr* R package. The CG-skew of a given sequence was defined as a proportion (C−G)/(C+G) and calculated with a sliding window width of 50 bp and a window increment step of 10 bp along the promoter sequence (Table S4). GC_3_ was calculated using CDSs and the R package *seqinr*. The slope of the GC_3_ gradient was estimated using linear regression between GC_3_ and the position relative to the first coding nucleotide (ATG) based on the first 1000 bp of a spliced transcript sequence. Genes were divided into GC_3_-poor and GC_3_-rich sets using 10% and 90% quantiles of GC_3_. DNA duplex stability was estimated using PromPredict [69] in a 15 bp sliding window. All manipulations were performed using bedtools and custom R and C scripts. A TFBS search was performed using the TRANSFAC database and MATCH [82].

### 4.5. Genome Visualization

The genomic sequences, tracks with gene models, predicted TSS, and RNA-seq coverage data were deposited at the Persephone genome viewer at https://web.persephonesoft.com (accessed on 31 January 2022). The choice of using this visualization solution was based on its ability to align and analyze genomic sequences in real time. Persephone is a state-of-the-art genome browser specifically designed to show and compare multiple sequences and genetic maps on one screen (see also help files at https://help.persephonesoft.com, accessed on 31 January 2022). The aligned maps can be linked using common markers, orthologous gene pairs, or regions of sequence similarity. The sequence maps can be aligned at a specific zoom level by running a real-time BLASTn search that visualizes structural variations by displaying identical sequence regions with highlighted nucleotide substitutions and indels.

## 5. Conclusions

This work is the first genome-wide prediction of TSS in genomes larger than 10 Gbp. Ancient origin, massive genome size, not associated with recent polyploidization or duplication, and extensive gene families (with higher copy number than in most angiosperms) distinguish conifers from other plants. The predicted TSSs and their putative promoter regions provide the basis for future experimental verification and present a valuable resource for better understanding gene regulation and investigating the evolutionary relationships between gymnosperm and angiosperm clades. Identification of TSS can find its implementation in genetic-assisted breeding and genome editing, providing opportunities for more precise mapping and the targeting of SNPs in functional genomic regions and quantitative trait loci associated with adaptive traits, such as growth rate, cold- and drought-resistance, and resistance to pathogen invasion.

We predicted promoter regions for several conifer species using computational strategies based on the expectation-maximization and neural network classification method utilized by the TSSPlant algorithm. The predictions were ranked using the probabilistic distributions of UTR lengths in model plants. The predicted TSSs were assessed using the profiles of standard free energy of a DNA duplex and the distribution of a CG-skew, both of which showed peaks around putative TSS positions and near the TBP binding site. The positional distributions of TFBS for several abundant transcription factor families also support the predicted promoters.

## Figures and Tables

**Table 1:** Summary of genome assemblies and annotations for four conifer species.

Assembly and Annotations Parameters	*Picea abies* [43]	*Picea glauca* [44]	*Pinus taeda* [45]	*Larix sibirica* [46]
Estimated genome size, Gbp	19.57	15.79	20.15	12.03
Assembly length, Gbp	12.30	25.47	22.10	12.34
Scaffold N50, Kbp	4.869	34.405	107.821	6.443
GC content, %	38.81	37.08	38.06	35.41
Repeat content, %	70.0		81.8	65–80 *
Total predicted gene models	58,587	103,694	36,732	50,163 *
Average CDS length, bp	287.21	283.56	419.81	291.01 *
Average intron length, bp	997.94	642.73	1146.12	351.13 *
Maximum intron length, bp	68,268	44,113	568,968	10,152 *
RNA/RefSeq supported genes *	10,434	16,839	9260	23,077
Predicted TSS positions *	25,889	44,651	22,633	62,420
Unique TSSs filtered by 5′-UTR distribution *	10,367	16,629	9149	23,016

* authors’ data.

## Data Availability

The datasets generated during the current study are available from the corresponding author on request.

## Supplementary Materials

The following supporting information can be downloaded at: https://www.mdpi.com/article/10.3390/ijms23031735/s1.
