# Supplementary material for: Genome-Wide Prediction of Transcription Start Sites in Conifers
Source: Int J Mol Sci. 2022 Feb 3;23(3):1735. doi: 10.3390/ijms23031735 (PMC8836283; doi:10.3390/ijms23031735)
Supplement: Supplementary file 1 [file ijms-23-01735-s001.zip › ijms-1555730-supplementary.pdf]

Supplementary Materials

Genome-wide prediction of transcription start sites in conifers

Eugenia I. Bondar[1,2], Maxim Troukhan[3], Konstantin V. Krutovsky[1,4-8], Tatiana V. Tatarinova[8-11]

[1] Laboratory of Forest Genomics, Institute of Fundamental Biology and Biotechnology, Siberian Federal University, 660036 Krasnoyarsk, Russia

[2] Laboratory of Genomic Research and Biotechnology, Federal Research Center "Krasnoyarsk Science Center," Siberian Branch, Russian Academy of Sciences, 660036 Krasnoyarsk, Russia

[3] Persephone Software LLC, 91301 Agoura Hills, CA, USA

[4] Department of Forest Genetics and Forest Tree Breeding, Georg-August University of Göttingen, 37077 Göttingen, Germany

[5] Center for Integrated Breeding Research, Georg-August University of Göttingen, 37075 Göttingen, Germany

[6] Laboratory of Population Genetics, N.I. Vavilov Institute of General Genetics, Russian Academy of Sciences, 119333 Moscow, Russia

[7] Scientific and Methodological Center, G. F. Morozov Voronezh State University of Forestry and Technologies, 394087 Voronezh, Russia

[8] Department of Genomics and Bioinformatics, Institute of Fundamental Biology and Biotechnology, Siberian Federal University, 660074 Krasnoyarsk, Russia

[9] Department of Biology, University of La Verne, 91750 La Verne, CA, USA

[10] Functional Genomics Group, N. I. Vavilov Institute of General Genetics, Russian Academy of Sciences, 119333 Moscow, Russia

[11] A.A. Kharkevich Institute for Information Transmission Problems, Russian Academy of Sciences, 127051 Moscow, Russia

Table S1. Resources used in the study.

Resource	Source	Identifier or filename
**Data**		
P. taeda genome assembly and annotation	treegenesdb.org	Pita.2_01.fa<br>Pita.2_01.gff
P. abies genome assembly and annotation	ConGenIE FTP on plantgenie.org	Pabies1.0-genome.fa.gz<br>Pabies1.0-HC.gff3<br>Pabies1.0-MC.gff3
P. glauca genome assembly and annotation	ConGenIE FTP on plantgenie.org	PG29-v3.fa<br>manualannotations-PG29V3.gff3<br>PG29v3-renamedID_1000nt.gff
L. sibirica genome assembly	NCBI GenBank	NWUY0000000000
P. glauca TSA	NCBI GenBank	GCHX00000000
P. glauca TSA	NCBI GenBank	GCZO00000000
P. glauca TSA	NCBI GenBank	GFBZ00000000
P. glauca TSA	treegenesdb.org	Pagl_TSA.fasta
P. glauca ESTs (313110 entries)	NCBI GenBank	
P. glauca ESTs	treegenesdb.org	Pagl_EST.fasta
P. abies ESTs (14345 entries)	NCBI GenBank	
P. abies putative unique transcripts	Chen et al., 2012	DRYAD DOI 10.5061/dryad.ds2gp
P. abies Trinity transcripts assembly	ConGenIE ftp on plantgenie.org	trinity.minKmer10.validated.fna.gz
P. taeda Sanger and 454 ESTs	PineDB Version 1.0	t3352.454.sanger.seqclean.newblertrim.tgz (http://bioinfolab.muohio.edu)
P. taeda ESTs (328662 entries)	NCBI GenBank	
P. taeda ESTs	treegenesdb.org	Pita_EST.fasta
A. thaliana genome annotation	arabidopsis.org	TAIR10_GFF3_genes.gff
A. thaliana promoter sequences	arabidopsis.org	TAIR10_upstream_1000_ translation_start_20101028.txt
O. sativa genome annotation	NCBI GenBank	GCF_001433935.1
S. bicolor genome annotation	NCBI GenBank	GCF_000003195.3
P. trichocarpa genome annotation	NCBI GenBank	GCF_000002775.4

Resource	Source	Identifier or filename
**Software and Algorithms**		
bedtools	Quinlan laboratory, University of Utah	bedtools.readthedocs.io
Hisat2	Johns Hopkins University	ccb.jhu.edu/software/hisat
TSSPlant	Computational Bioscience Research Center (CBRC), King Abdullah University of Science and Technology (KAUST)	http://www.cbrc.kaust.edu.sa/download
PromPredict	Molecular Biophysics Unit, IISC	nucleix.mbu.iisc.ernet.in/prompredict
TRANSFAC	QIAGEN GmbH	https://genexplain.com
MATCH	QIAGEN GmbH	https://genexplain.com
MEME suite 5.3.3	National Institutes of Health	https://meme-suite.org/meme
R package stringr	CRAN	cran.r-project.org/web/packages/stringr
R package seqinr	CRAN	cran.r-project.org/web/packages/seqinr
R package ggplot2	CRAN	cran.r-project.org/web/packages/ ggplot2
R package data.table	CRAN	cran.r-project.org/web/packages/data.table
R package *ggsci*	CRAN	cran.r-project.org/web/packages/ggsci
R package Biostrings	bioconductor.org	10.18129/B9.bioc.Biostrings
R package reshape2	CRAN	cran.r-project.org/web/packages/reshape2

Table S2. Independent two-sample Mann Whitney U Test (two-sample Wilcoxon rank-sum test) results for GC3-poor and -rich genes (CDS sequences).

Species	W	*p*-value
Siberian larch (*Larix sibirica*)	438206	$< 2.2\times10^{-16}$
Norway spruce (*Picea abies*)	180196	$< 2.20\times10^{-16}$
White spruce (*Picea glauca*)	286581	6.09×10^{-12}
Loblolly pine (*Pinus taeda*)	539415	$< 2.20\times10^{-16}$

Table S3. Number of promotors containing TATA-box or CA initiator motif or both TATA and CA.

Species	Total promoters	TATA (%)	CA (%)	Both (%)	Ratio TATA to TATA with CA
L. sibirica	22 291	1 295 (5.8)	10 262 (46.0)	664 (3.0)	2.0
P. abies	10 120	640 (6.3)	4 965 (49.1)	331 (3.3)	1.9
P. glauca	16 255	911 (5.6)	7 772 (47.8)	460 (2.8)	2.0
P. taeda	9 064	713 (7.9)	4 707 (51.9)	426 (4.7)	1.7
A. thaliana	27 100	1 472 (5.4)	14 380 (53.1)	911 (3.4)	1.6

Table S4. Parameters used for running HISAT, BLAST, CG-skew analysis and selection of the best 5' UTR prediction.

Tool	Parameters
HISAT2	hisat2 -x <genome_index> -f -U <RNA_&_EST> --no-unal -p 20 --no-hd -S out.sam
BLAST	blastp -query proteins.faa -db Refseq_plant -outfmt "6 qacc sacc stitle evalue length pident qstart qend sstart send" -num_threads 20 -max_target_seqs 5
5'-UTR selection	$f(x) = \frac{1}{\Gamma(k)\theta^k} x^{k-1} e^{-\frac{x}{\theta}}$<br><br>$\theta$ = *variance / mean*<br>k = *mean /* θ<br><br>*Density = dgamma(TSS$lenght, shape = k, scale = theta)*
CG-skew	$CGskew_i = (nC_i - nG_i)/(nC_i + nG_i)$,<br><br>$nC_i$, nG_i – number of C and G nucleotides in a window *i*,<br>The sliding window was 50 bp wide, and a window increment step of 10 bp.

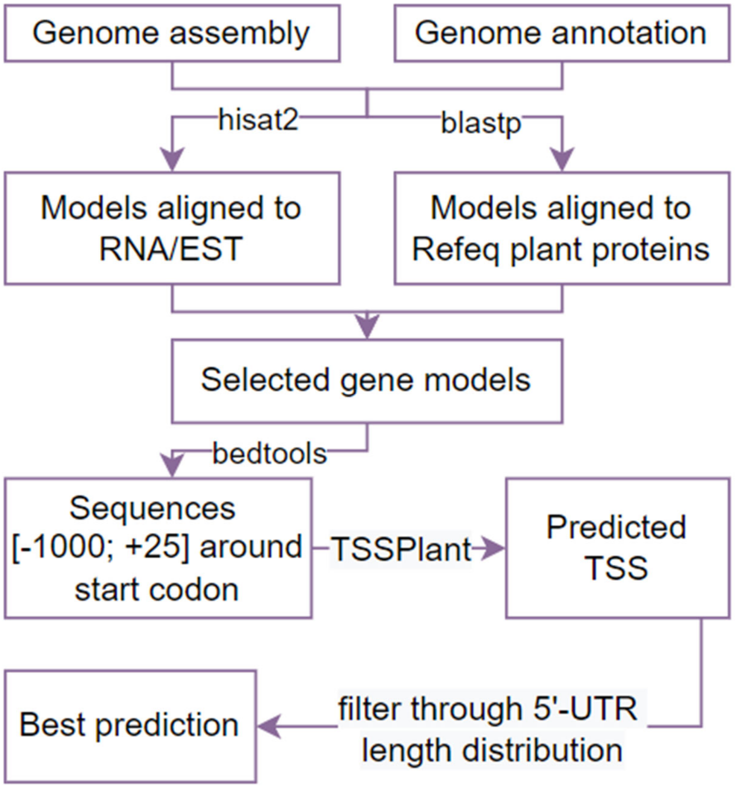

Figure S1. The workflow for the genome-wide TSS identification.

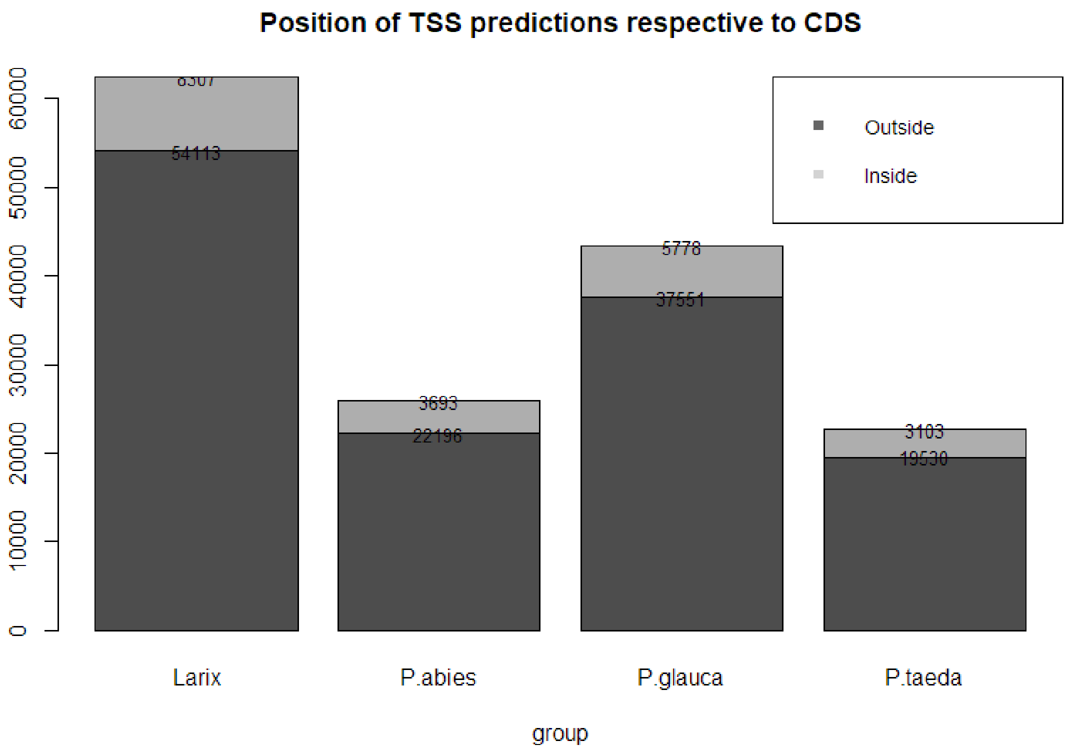

Figure S2. The number of predicted TSSs in *L. sibirica, P. abies, P. glauca* and *P. taeda* (before filtering through typical 5'-UTR length distribution) that intersect their respective gene models.

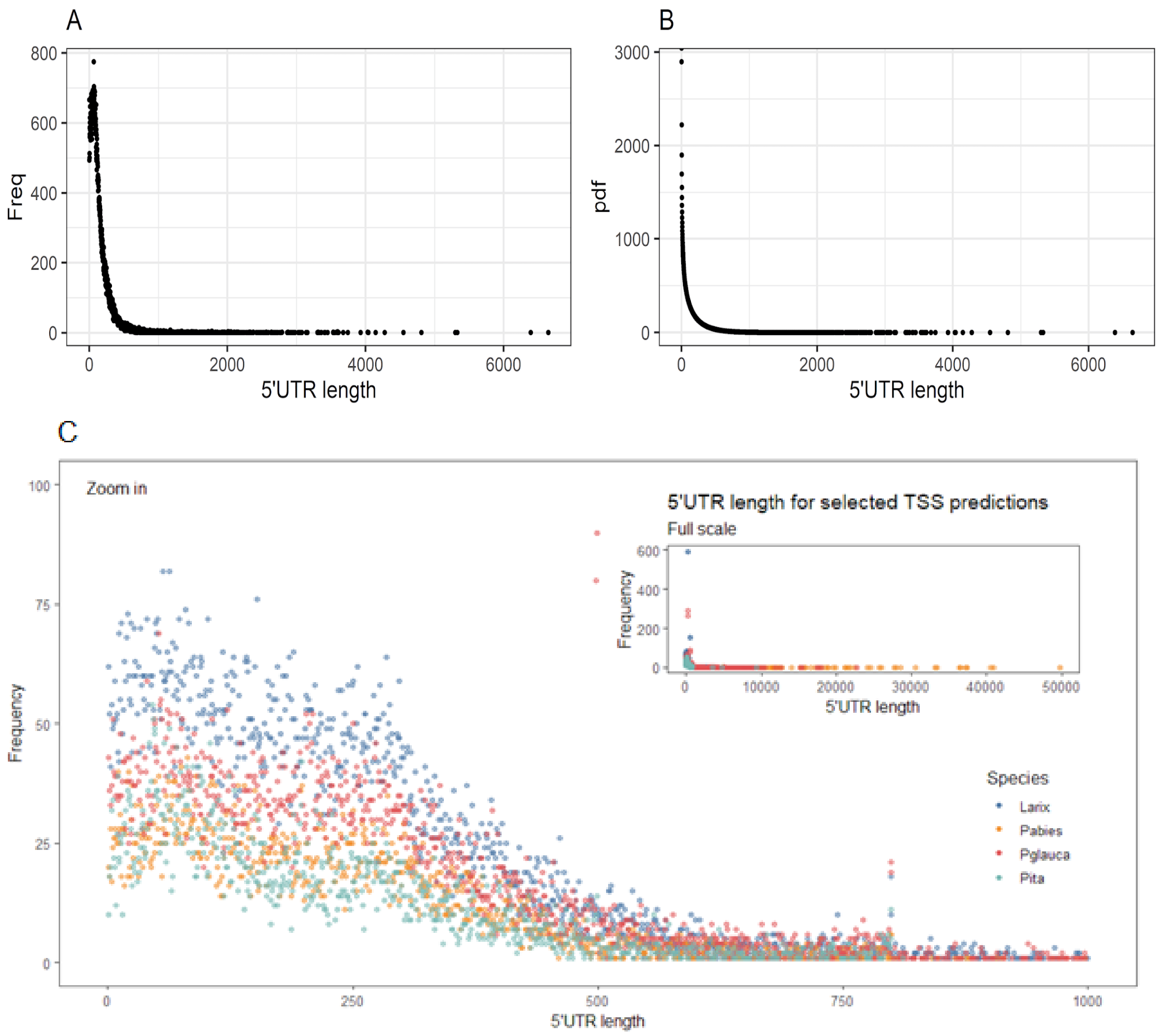

Figure S3. A and **B**: the distribution of 5'UTR lengths based on *A. thaliana, P. trichocarpa, O. sativa* and *S. bicolor*; **C:** the distribution of 5'UTR lengths in four conifer species.

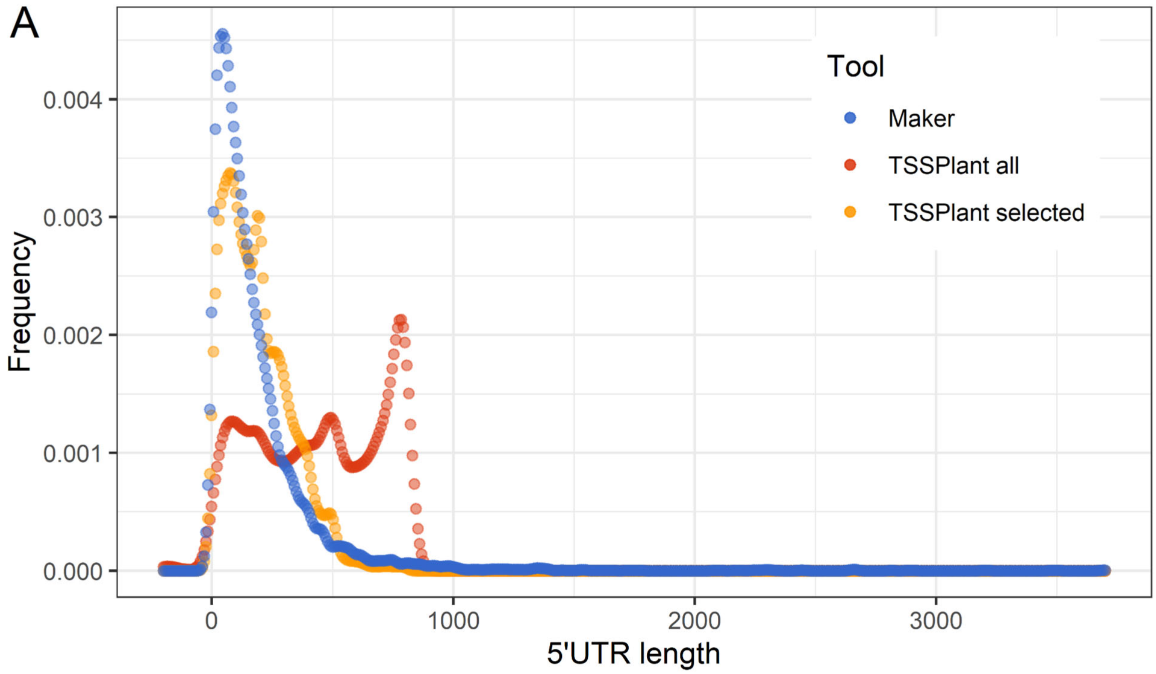

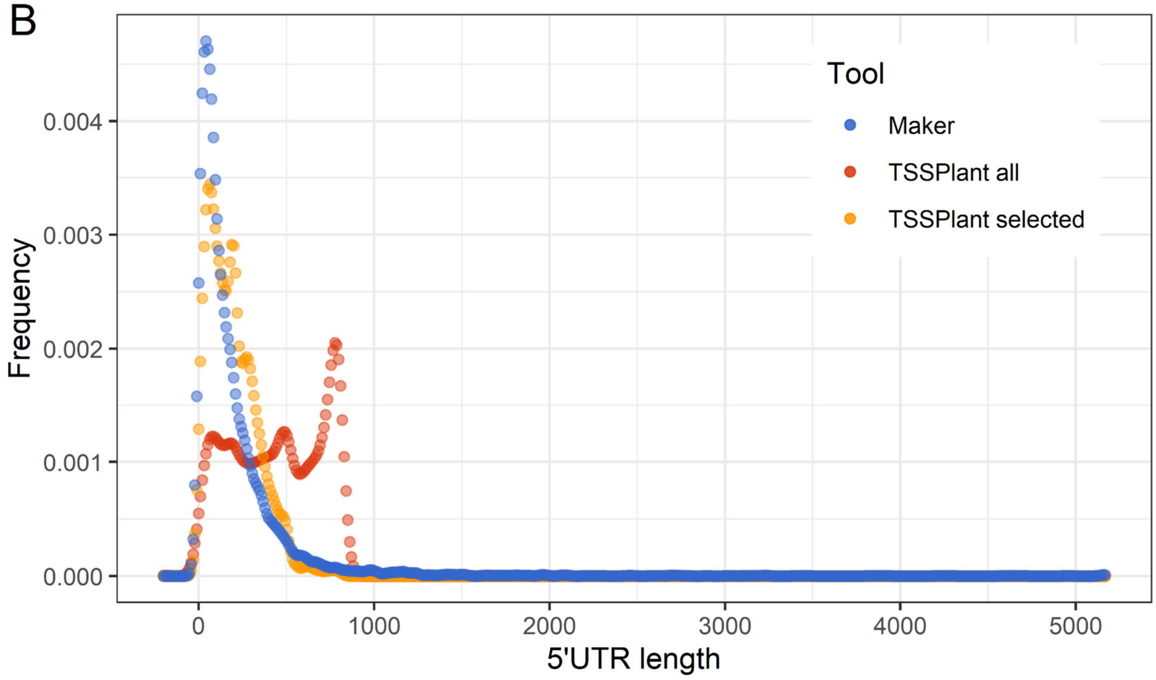

Figure S4. Comparison of 5'UTR length predicted by the Maker pipeline and by TSSPlant in the genome of *L. sibirica* (**A**) and *P. glauca* (**B**).

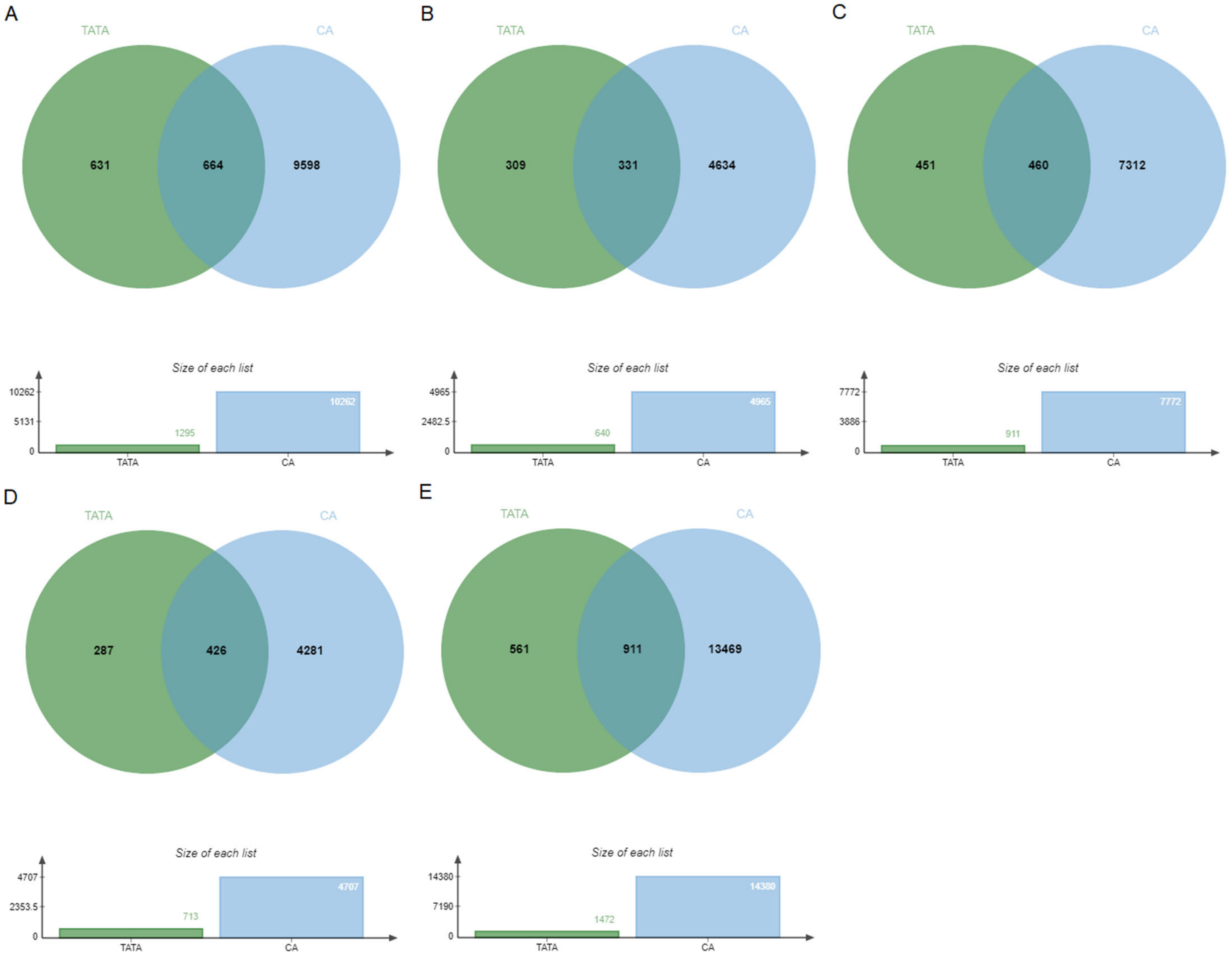

Figure S5. Number of promotors containing TATA-box (green circle) or CA (blue circle) or both motifs. **A** – *L. sibirica*, **B** – *P. abies*, **C** – *P. glauca*, **D** – *P. taeda*, **E** – *A. thaliana*.

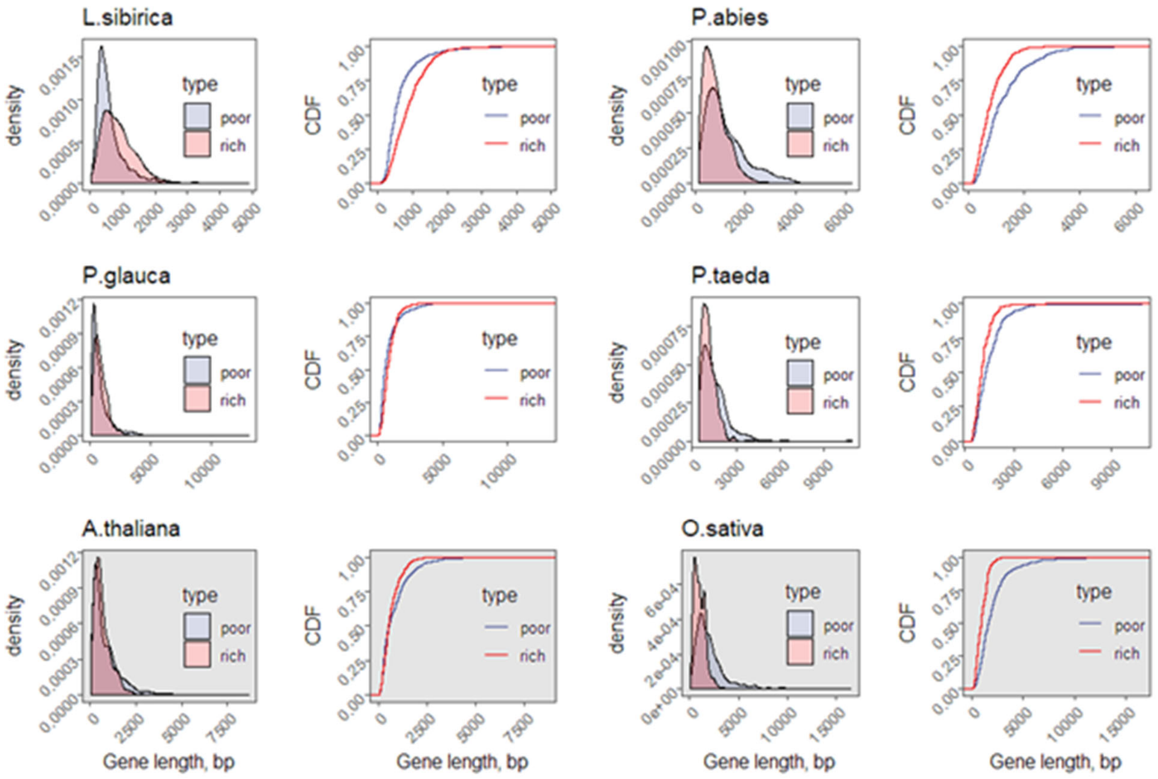

Figure S6. Gene length distribution and cumulative distribution (CDF) in GC3-poor and GC3-rich genes for four conifer species (*L. sibirica, P. abies, P. glauca* and *P. taeda*) and two model plant species (*A. thaliana* and *O. sativa*)
